# Supplementary material for: Barriers to adaptation of environmental sustainability in SMEs: A qualitative study
Source: PLoS One. 2024 May 16;19(5):e0298580. doi: 10.1371/journal.pone.0298580 (PMC11098482; doi:10.1371/journal.pone.0298580)
Supplement: Attachment — Submitted filename: PONE-D-23-22784.docx [file pone.0298580.s002.docx]

**Unlocking the Barriers: Exploring the Impediments to Environmental Sustainability in Developing Country SMEs**

#### Manuscript ID: PONE-D-23-22784

Dear authors,

Thank you for the opportunity to review this very interesting piece. While I believe your work is worth publishing, there are several issues that I would like to address prior to this.

**First Extensive editing of the English language and style is required.**

The title is confusing Please re-write.

**Abstract**

Please concise abstract of the study.

**Introduction**

- Set the stage for context, and history, and summarize what you’re going to do. Leave the reader with some impression as to the importance of the topic.
- There are many writing errors such as“ (Even though the research on environmental sustainability is growing, there is gap in how SMEs can achieve environmental sustainability especially in context of recourse constraint developing country.”) What is this? Where is in/of?
- Why there is a need to explore the barriers and limitations of a developing country? There should be a strong background please pay attention to this part.

**Literature review**

- You used too many headings. Please read some articles to get a paper writing idea.
- The literature review in the study should be like a logical story that tells the readers about the gap that you intend to fill. What does the existing literature tell us? What do we know and don't know based on literature?

**Methods and Results**

- In the data collection you do not need to add the aim of the study: (The present study aimed to investigate the barriers to sustainable practices adoption in small and medium-sized enterprises (SMEs) in Balochistan, Pakistan.) You should discuss this in the introduction.
- I appreciate the results description, but again in the results, there are too many headings. I suggest you read some papers to follow the structure.

**Discussion and Conclusion**

**-** The discussion must be rewritten considering the findings. The author should have a higher deepness in the discussion, making the linkage to the results of other papers and the theory.

- Overall manuscript has replication please avoid it when revising.

- Spell out the full term at its first mention, indicate its abbreviation in parenthesis, and use the abbreviation from then on. Such as Small and Medium-sized Enterprises (SMEs).
