# Supplementary material for: Barriers to adaptation of environmental sustainability in SMEs: A qualitative study
Source: PLoS One. 2024 May 16;19(5):e0298580. doi: 10.1371/journal.pone.0298580 (PMC11098482; doi:10.1371/journal.pone.0298580)
Supplement: Attachment — Submitted filename: Response to Reviewers PONE-D-23-22784.R1.docx [file pone.0298580.s003.docx]

**RESPONSE SHEET**

**Manuscript ID:  PONE-D-23-22784.R1**

Dear Editor,

We would like to thank you for considering our manuscript submitted to PLOS ONE Journal for possible publication and providing us an opportunity to revise and resubmit our manuscript based on the reviewers’ comments. We appreciate and acknowledge the inputs offered by reviewers that have helped us to improve the presentation and quality of the manuscript.

In the report below we have described the actions we have taken in response to each query raised by reviewers. We hope that you will find the changes we have made to be sufficient for acceptance of the paper.

With regards,

On-behalf of all the co-authors

Action Taken Report

Dear Reviewers,

We would like to express our sincere gratitude to you for devoting your quality time spent on reviewing our manuscript and offering us detailed inputs which have been extremely helpful in shaping our manuscript.

We hope you will appreciate our sincere efforts in revising the manuscript. Below, we present our responses and actions in tabulated form.

|  | Editor Comments | Author's Response |
| --- | --- | --- |
| 1 | Please ensure that your manuscript meets PLOS ONE's style requirements, including those for file naming. The PLOS ONE style templates can be found at  <https://journals.plos.org/plosone/s/file?id=wjVg/PLOSOne_formatting_sample_main_body.pdf> and  <https://journals.plos.org/plosone/s/file?id=ba62/PLOSOne_formatting_sample_title_authors_affiliations.pdf> | Thank you. We have followed the formatting guidelines of PLOS ONE. |
| 2 | You indicated that ethical approval was not necessary for your study. We understand that the framework for ethical oversight requirements for studies of this type may differ depending on the setting and we would appreciate some further clarification regarding your research. Could you please provide further details on why your study is exempt from the need for approval and confirmation from your institutional review board or research ethics committee (e.g., in the form of a letter or email correspondence) that ethics review was not necessary for this study? Please include a copy of the correspondence as an ""Other"" file. | This study is quantitative, the authors conducted interviews with managers and owners of SMEs to explore their awareness and attitudes toward sustainability practices in business. Furthermore, the study did not involve physical testing or direct contact with respondents. Consequently, there is no possibility of harm—whether mental or physical—to the participants. This absence of risk enables the authors to conduct the study without implementing any precautionary protocols, distinguishing it from qualitative studies in medical research.  Further, we have provided an official letter from the review board stating that no ethical approval was required for this research paper. |
| 3 | In your Data Availability statement, you have not specified where the minimal data set underlying the results described in your manuscript can be found. PLOS defines a study's minimal data set as the underlying data used to reach the conclusions drawn in the manuscript and any additional data required to replicate the reported study findings in their entirety. All PLOS journals require that the minimal data set be made fully available. For more information about our data policy, please see http://journals.plos.org/plosone/s/data-availability.  Upon re-submitting your revised manuscript, please upload your study’s minimal underlying data set as either Supporting Information files or to a stable, public repository and include the relevant URLs, DOIs, or accession numbers within your revised cover letter. For a list of acceptable repositories, please see http://journals.plos.org/plosone/s/data-availability#loc-recommended-repositories. Any potentially identifying patient information must be fully anonymized.  Important: If there are ethical or legal restrictions to sharing your data publicly, please explain these restrictions in detail. Please see our guidelines for more information on what we consider unacceptable restrictions to publicly sharing data: http://journals.plos.org/plosone/s/data-availability#loc-unacceptable-data-access-restrictions. Note that it is not acceptable for the authors to be the sole named individuals responsible for ensuring data access.  We will update your Data Availability statement to reflect the information you provide in your cover letter. | Thank you. We have uploaded data set underlying the results of this study. |
| 4 | Please ensure that you include a title page within your main document. You should list all authors and all affiliations as per our author instructions and clearly indicate the corresponding author. | Thank you. We have uploaded the title page as per author's instructions. |
| 5 | Please include your full ethics statement in the ‘Methods’ section of your manuscript file. In your statement, please include the full name of the IRB or ethics committee who approved or waived your study, as well as whether or not you obtained informed written or verbal consent. If consent was waived for your study, please include this information in your statement as well. | Thank you. We have included a statement indicating that ethical approval was waived by the Institutional Review Board, as shown below:  Page 6, Lines 35-38  This research adhered to ethical guidelines, securing respondent consent prior to interviews. Additionally, the authors sought ethical approval from the institutional review board (IRB). However, the IRB returned the application with the recommendation that no ethical review is necessary for this research study. |

|  | Reviewer 1 Comment | Author's Response |
| --- | --- | --- |
| 1 | The paper is well-written and provides a clear and concise overview of the study. It is well-organized and easy to read, and it effectively highlights the key findings and contributions of the research. However the paper is written in a report format. | Thank you for your comment. We have followed your instructions to improve our article. |
| 2 | Add a flowchart/diagram representing the process study made. | Thank you. We have included a figure representing the flow of the research process on page 7, shown in figure 2. |
| 3 | Supporting results in form of tabular data/graphs can be added. | Thank you for your comment. We have incorporated the results in tabular form on pages 9 and 10, in Tables 2 and 3. |
| 4 | In the findings section, consider providing more specific examples or details to support the key findings. | Thank you for your comment. We have provided specific examples in support of our findings. |
| 5 | In the practical/implications section, consider discussing the specific implications of the research for SME owners/managers, customers, and government policymakers. | Thank you for your comments. We have improved the research implications on page 19, lines 19-41.  Page 19 Lines 19-41.  The study provides significant implications for SMEs owners, managers, government, and environmental agencies. First, it is utmost important for both owners and workers to have access to education regarding environmental initiatives and their advantages. Closing the knowledge gap through workshops and training programs is essential, not only for aware SMEs but also to make them stick to eco-friendly protocols. Additionally, government agencies and environmental NGOs should advocate for increased government support and incentives as financial limitations pose a barrier. Second, SMEs should view ISO certification not as a requirement but also as a strategic tool that provides an advantage in accessing international markets. SMEs should engage with customers to raise awareness about the benefits of eco-friendly practices, which can help address customers’ concerns about increased prices. Third, SMEs should position themselves as environmentally friendly organizations that open opportunities for financial support mechanisms such as environmental initiative grants and loans. Fourth, managers should develop CSR strategies that align with SMEs priorities, which create shared value and enhance reputation. Fifth, managers should collaborate with other environmental agencies and establish networks with NGOs and governmental agencies to amplify the impact of sustainability initiatives. Managers should also explore the opportunities for adaptation of climate friendly technologies and processes. Sixth, government agencies should take initiatives to foster a mindset among SMEs owners by emphasizing how sustainability aligns with long term profitability. Finally, government agencies should develop policies to monitor and evaluate environmental initiatives and performance of SMEs, which ensures improvement and adaptation to environmental initiatives. These research implications contribute to long term success and sustainable development of SMEs. |

|  | Reviewer 2 Comment | Author's Response |
| --- | --- | --- |
| 1 | Thank you for the opportunity to review this very interesting piece. While I believe your work is worth publishing, there are several issues that I would like to address prior to this. | Thank you for your comments. We have improved the article following your instructions. |
| 2 | First Extensive editing of the English language and style is required. | Thank you for your comment. We have proofread our article. |
| 3 | The title is confusing. Please re-write. | Thank you for your comment. We have changed the title as follows:  Barriers to adaptation of environmental sustainability in SMEs: A qualitative study |
| 4 | Abstract: Please concise abstract of the study. | Thank you for your comment. We have improved the abstract of the study, as given below:  Abstract  This study examines the antecedents of environmental sustainability in small and medium enterprises (SMEs) of a developing country and explores the specific internal and external factors for environmental sustainability. The study focused on SMEs in Balochistan, Pakistan, utilizing convenience and purposive sampling techniques to select a sample size of 30 SMEs. In-depth qualitative interviews were conducted using a semi-structured questionnaire. The results of the study revealed that lack of finance and education are major barriers to recognizing and addressing environmental sustainability issues, along with the lack of government support and regulations to ensure compliance with environmental safety laws, hence leading to low concern for sustainability practices among SMEs. Awareness and attitude of SME owners/managers, along with customer demand and government policies, influence the adoption of environmental sustainability practices. Overcoming financial constraints and promoting cooperation among stakeholders are key to fostering sustainable practices in SMEs. This research makes an important contribution to the sustainable management literature by providing new and in-depth insights into the barriers that impede environmental sustainability in SMEs of developing countries. |
| 5 | Introduction  - Set the stage for context, and history, and summarize what you’re going to do. Leave the reader with some impression as to the importance of the topic.  - There are many writing errors such as “Even though the research on environmental sustainability is growing, there is gap in how SMEs can achieve environmental sustainability especially in context of recourse constraint developing country.” What is this? Where is in/of?  - Why there is a need to explore the barriers and limitations of a developing country? There should be a strong background please pay attention to this part. | Thank you for your comments. We have improved the introduction by following your suggestions.  Page 1, Lines 30-36  SMEs have not received significant attention in the ongoing global discussion about sustainable development [4, 5]. It is evident that large organizations impact environment. It is also crucial to recognize the role of SMEs as well. SMEs impact on environment become clearer when we consider their collective impacts. Globally, over 95% of businesses are SMEs [6]. Although each SME may have little impact, the cumulative effect of SMEs can be substantial due to their large number [4]. It is often mentioned that SMEs are responsible for major pollution [7].  Page 1, Lines 40-45; Page 2, Lines 1-7  Researchers have been supporting the adaptation of sustainability practices [11, 3]. However, literature has not adequately addressed the sustainability issues faced by SMEs in developing countries as it has with developed ones [12]. This situation has been widely acknowledged in the literature [4, 5]. Wang et al. [10] shed light on the importance of sustainability initiatives in developing countries and the unique barriers and motivational factors. Similarly, Jabbour et al., [4] emphasized the significance of sustainability in developing countries and the need to study strategies for achieving sustainable practices. Additionally, Purwandani & Michaud [13] recognized the necessity to delve into sustainability matters within SMEs as it impacts business performance and presents new market opportunities.  Page 2, Lines 14-27  In this study we address the gap by exploring the internal and external factors that contribute to achieve the environmental sustainability in SMEs of Pakistan. The present study recognizes the existence of environmental alerts in Pakistan, particularly in Balochistan that comprise great environmental concern and weak governance [15, 16, 17]. Hence, this research significantly contributes to the discipline of sustainability. Further, the research framework of this study encompasses environmental orientation, responsible environmental management, and eco-friendly practices, which are grounded on strategic competencies such as pollution prevention, product stewardship, and sustainable development of natural resource-based view (NRBV) theory [18, 19]. Therefore, this study provides comprehensive understanding of internal and external dimensions of SMEs that need to be nurtured to improve environmental sustainability. While extending the previous research Baah et al. [18], this research provides new insight for top management, policymakers, governmental and non-governmental organizations (NGOs) for achieving environmental performance in SMEs of developing countries. |
| 6 | Literature review  - You used too many headings. Please read some articles to get a paper writing idea.  - The literature review in the study should be like a logical story that tells the readers about the gap that you intend to fill. What does the existing literature tell us? What do we know and don't know based on literature? | Thank you for your comment. We have rewritten the literature review and incorporated your instructions on page 2, lines 44-47; page 3, lines 1-50; page 4, lines 1-21; page 5, lines 1-45; page 6, lines 1-27. Page 2, Lines 44-47; Page 3, Lines 1-50; Page 4, Lines 1-21; Page 5, Lines 1-45; Page 6, Lines 1-27 SMEs prioritize survival and cost reduction that may not align with environmental sustainability [25]. The relationship between environmental sustainability and economic outcomes is complex and influenced by multiple factors. However, engaging in environmentally efficient practices can lead to long-term benefits and increased revenue through recycling, reuse and reduce [26]. Hence, it raised question whether eco-efficiency could increase profits for SMEs [27]. Researchers suggest that adaptation of sustainable practices and ecological partnerships can lead to financial success for SMEs [28]. Moreover, Hossain et al. [29] conducted a systematic literature review of articles published during 2009–2020 and identified 87 drivers of environmental sustainability and categorized them under internal and external dimensions. Researchers suggested that SMEs should focus on internal and external factors for implementation of sustainable practices. Hence, researchers identified internal and external barriers suitable to the context of this study. Table 1 summarizes the barriers and opportunities for SMEs. In reality, adopting sustainability requires resources, however, SMEs already facing several challenges [30]. On the other side, SMEs strive to gain flexibility and adaptability [31], unfortunately SMEs often lack the resources to implement sustainable practices [10]. Researchers argue that factors such as time constraints, financial limitations, inadequate training, information access and a lack of environmental management are hindering SMEs in practicing environmental sustainability [13, 32]. A limited number of staff and routine work leave no time for learning and implementing sustainability measures [32]. Hiring professionals to train existing employees can help, however, financial constraints restrict SMEs to invest in knowledge and ability enhancement. Another aspect is employee or manager reluctance towards green innovation due to tight schedule, lack of experience or latest knowledge [33]. Environmental practices require pro-environmental behavior of employees [34]. To address this problem, companies should establish a network and enhance their relationship with external partners [35], such as universities, to provide cost-effective training for their staff. Establishing short-term objectives may facilitate the achievement of long-term goals, allowing SMEs to foster internal sustainability. The limited financial capital of SMEs creates a significant constraint in SMEs ability to invest in environmental sustainability practices. The impact of these investments on profits remains a lingering question. Despite the potential benefits, SMEs may struggle to sustain the financial burden of implementing environmental sustainability measures [36]. Consequently, SMEs can apply for grants and loans from government agencies and financial institutions to alleviate financial constraints, which, in return, help them to bring sustainability in process and increase revenue in long term. However, lack of financial support from government and financial institutions and formal lengthy procedures creates hindrances in adopting sustainable practices [13, 37]. Despite the awareness and willingness of owners and managers to embrace environmental sustainability, financial constraints prevent them from environmental initiatives or involving third parties [38]. Moreover, the decision to adopt environmental sustainability practices in SMEs lies with the owners and managers, who act as the decision-makers. Their perception of the benefits associated with pro-environmental behavior is a key factor in determining whether they choose to invest in such practices [34]. A positive attitude towards environmental sustainability is essential for owners and managers to initiate such practices in their firms [38, 39]. Manager’s age determines the adaptation of sustainable practices and concerns for environmental performance [40]. Education and green entrepreneurial orientation also play a significant role in adopting sustainable practices in SMEs [35]. SMEs are mostly governed by their owners and family members hence they align their resources with their priority, and survival is the topmost priority. Thus, sustainability may be considered on priority in SMEs when owners, family member or managers have environmental instincts and can foresee that bringing sustainability is new way of survival. On the other hand, the interests of stakeholders play a significant role in firm's approach towards environmental sustainability at different levels within the business [41]. Stakeholders often prioritize financial sustainability over social and environmental sustainability, as long as they receive some sort of return [42]. Large organizations can establish long-term partnerships with stakeholders and the resources to influence through marketing strategies. Albeit SMEs find it difficult to maintain the relationships in long-term and often have shorter-term relationships with stakeholders, thus making it difficult for SMEs to change their perceptions [25]. SMEs are supposed to have a responsibility to the community, customers, and employees they serve [37, 38, 43]. Jamali et al. [44] argue that SMEs operate on a personal level and are closer to their stakeholders due to work-family issues, trust, and employee retention, among other factors. Moreover, when owners see market forces and customers direction towards eco-friendly processes and products, they may shift their focus on sustainability. Researchers claim that owners may not consider sustainability issues until they face a problem and are not motivated to do so as long as they are reaping profits [45]. **Table 1. Barriers and opportunities for SMEs.** **Barriers For SMEs** \| **Opportunities for SMEs**; - Insufficient knowledge among owners/managers or employees. - Limited workforce size. - Constrained financial resources. - Inadequate organizational capacity. - Restricted and short-term business operations integrated into daily activities. - Pessimistic belief that engaging in environmental sustainability practices might have a prolonged payback period. - Non-compliance or lack of adherence to government legislation. - Concerns about potential temporary cost increases for products, with owners fearing customer resistance to higher prices. - Different levels of awareness among owners/managers. - Diverse attitudes among owners/managers. - Reluctance of owners/managers to alter their perspectives on environmental sustainability practices. - Lack of awareness among stakeholders regarding the advantages of environmental sustainability practices compared to potential price increases. - Limited knowledge about technological advancements and their potential benefits for overall SME performance. \| - Implementation of laws and regulations mandating SMEs to adopt environmental sustainability practices. - Flexibility in altering the existing setup. - Proximity and strong ties with suppliers. - Commitment of owner management towards incorporating environmental sustainability practices. - Introduction of green innovative products. - Utilization of part-time employees to align with new practices. - Enhanced awareness about the benefits of environmental sustainability practices. - Influence from community expectations regarding environmental sustainability. - Increasing interest from investors in supporting and promoting environmental sustainability practices. **Environmental sustainability practices and firm performance** The incorporation of sustainable practices in SMEs is influenced by various factors [10, 13]. These factors include the motivation of entrepreneurs to adopt certain practices based on their perception of potential positive financial outcomes or their concerns about the consequences of not embracing eco-friendly approaches [46]. The actions taken towards sustainability can either positively or negatively impact the financial performance of SMEs. Some researchers suggested that implementing sustainability practices is considered an additional burden by SMEs [47], while others support that there is positive relationship between firm performance and environmental protection efforts [8, 36]. Environmental Management System (EMS) has proven to be highly beneficial for the sustainable development of SMEs. ISO 14001 offers certified guidelines to assist SMEs in reducing their environmental impact and achieving long term sustainability [48]. By implementing EMS, SMEs can lower energy consumption costs while also creating positive image in the eyes of customers, regulators, and the public. Furthermore, EMS enables SMEs to utilize latest green technology, which not only reduces costs and improves productivity but also minimizes their negative impact on the environment. This innovative approach to resource utilization, cost reduction and environmental preservation ultimately ensures the sustainability of SMEs [24, 49]. Additionally, when SMEs collaborate with businesses within their network and receive support from organizations, they become more environmentally innovative and effectively reduce costs [22]. This ability to leverage network advantages is crucial for SMEs. Subsequently, adoption of environmental practices allows SMEs to position themselves as excellent performers [50]. Researchers suggested that financial limitations were inversely related to the adoption of practices. However, it also revealed a correlation between access to capital and technological expertise with the implementation of sustainability measures regardless of company size [50]. Various approaches can be utilized to assess the impact of environmental sustainability practices on firm performance. These include enhancing stability, reducing emissions , improving eco-efficiency systems, conserving materials, energy and implementing ISO 14001 certification [9, 8, 24, 48]. Performance measures may involve creating environmentally friendly production processes and adopting innovative techniques. By adopting environmental sustainability practices firms can enhance their systems to minimize water usage, air pollution and toxic waste emissions [51]. Therefore, managers of SMEs who implement sustainability measures often believe in adopting innovative technologies [52]. The positive impact of effectively implemented EMS, on performance has been evident [48, 53]. Therefore, implementation of EMS leads to improved environmental performance, including reductions in water consumption and waste emissions. Previous studies found that the adoption of EMS practices provides benefits to SMEs [53]. Improvements in environmental performance and cost reduction go hand in hand. Eco-design plays a role in creating products with better recycling potential and improved performance. Furthermore, these eco-friendly designs are not only less harmful but also cost-effective to manufacture, leading to reduced expenses and improved firm performance [54]. When organizations strive to reduce pollution, it ultimately results in resource utilization and increased productivity. Sustainable practices like recycling and collaborating with suppliers contribute to cost reduction and enhanced efficiency. By utilizing metal scraps, waste materials, and recycled oil within SMEs operations along, with water recycling and purification measures, all contribute to cost reduction while simultaneously boosting profits. Furthermore, implementing environmental protection initiatives can influence firm image [54]. Firms’ performance heavily relies on their knowledge-based resources and the ability to effectively utilize these resources to gain advantages [52]. However, researchers argue that being an eco-friendly organization does not necessarily guarantee strong competitiveness in the market [50]. **Conceptual framework** This study proposes a research model to investigate sustainability in SMEs. Previous studies have examined how internal and external factors affect knowledge and attitudes toward environmentally conscious management practices [55]. This study included external and internal factors as independent variables, which encompasses suppliers, customers, and laws. These characteristics motivate and challenge management to adopt environmental sustainability initiatives. Stakeholder impact on SMEs is further influenced by environmental awareness and attitudes, which address top management understanding of the cost and benefits of being environmentally sustainable. These variables influence environmental sustainability practices, serving as dependent variable. Hence, external and internal factors influence the organization's attitude and understanding of environmental sustainability, which defines its policies, strategies, and systems for sustainable development. This process is influenced by owner-manager qualities, time, financial resources, and cost-benefit information about environmental sustainability. Without these criteria, SMEs may struggle to implement environmental sustainability initiatives. The effects of these factors need to be examined in the context of developing countries. Fig 1 depicts the research framework. ` **External** **Factors** **Environmental Awareness and Attitudes** **Environmental Sustainability Practices** **Internal** **Factors** **Fig 1. Conceptual framework.** |
| 7 | Methods and Results  - In the data collection you do not need to add the aim of the study: (The present study aimed to investigate the barriers to sustainable practices adoption in small and medium-sized enterprises (SMEs) in Balochistan, Pakistan.) You should discuss this in the introduction.  - I appreciate the results description, but again in the results, there are too many headings. I suggest you read some papers to follow the structure. | Thank you for your remarks. We have removed redundant sentences and headings. |
| 8 | Discussion and Conclusion  - The discussion must be rewritten considering the findings. The author should have a higher deepness in the discussion, making the linkage to the results of other papers and the theory.  - Overall manuscript has replication please avoid it when revising.  - Spell out the full term at its first mention, indicate its abbreviation in parenthesis, and use the abbreviation from then on. Such as Small and Medium-sized Enterprises (SMEs). | Thank you for your comment. We have improved the discussion and created a linkage of previous literature with our results on page 17, lines 3-17; page 18, lines 1-50; page 19, lines 1-15.  The study aimed to understand the level of awareness among owners and senior managers of SMEs regarding environmental sustainability issues. The results are aligned with strategic abilities of NRBV theory, such as pollution prevention, product stewardship, and sustainable development. NRBV provides support for the theme of this study, which encompasses environmental-orientation, responsible environmental management, and eco-friendly operations.  The qualitative analysis revealed that majority of SMEs owners or managers were not familiar with the term "ecopreneurship." This was primarily due to their low level of education, knowledge, or lack of concern for environmental issues. Researcher argues that environmental practices rely on equal involvement from all employees and lack of knowledge hinders their implementation [20]. In Balochistan, the shortage of electricity, water, law enforcement and mismanagement are well known [16]. Every respondent interviewed acknowledged this problem and agreed on the need to conserve these resources. However, the awareness was limited to owners and managers, and they faced difficulties in making their workers understand and address sustainability.  Most SMEs, except for a few that were ISO certified, did not consider environmental impact in their business practices. Firms that obtained ISO certification only did so to expand their international business, as certification was mandatory for international business dealings. These findings are consistent with previous literature suggesting that unawareness and lack of laws are major reasons for environmental harm [63]. When law enforcement is weak, people tend to harm the environment. However, strict regulations and the fear of punishment may prompt SMEs to make changes that help them understand the costs and benefits of their actions. Hence, external pressure such as regulatory compliance and legal certainty, is a primary motivator for firms to adopt environmental practices [18, 32]. Thus, it is important for customers, governments, and supportive organizations to work together to educate and encourage SMEs to adopt environmentally sustainable practices.  Sustainable practices play a critical role for businesses in boosting their market share. Researchers suggested that sustainable practices offer benefits such as enhanced market position, increased customer base, and competitive edge [64]. In line with previous studies, this study identified that SMEs that are ISO-certified believe they must be environmentally sustainable to capture international customers [37, 38, 43, 44]. Further, external factors, such as customers, can act as both motivators and obstacles for SMEs in terms of adopting environmentally sustainable practices. However, local customers who are price-sensitive may be hesitant to accept the potential price increases that come with environmentally sustainable practices. Meanwhile, SMEs in Balochistan province face further hurdles as their supply chains come from poor backgrounds, lacking financial resources and government support, forcing them to prioritize their livelihood over environmental sustainability [65].  The adoption of environmental sustainability practices by SMEs is a subject of debate due to their limited financial capital. It remains unclear whether such investments will result in increased profits or whether SMEs may face financial challenges when engaging in such practices [36]. On the other hand, applying for grants and loans from government agencies, energy service companies, or non-government organizations can help SMEs overcome financial constraints and pursue environmental sustainability [66]. However, some studies have suggested that the willingness to adopt environmental sustainability practices has nothing to do with the economics or size of the firm but rather depends on the awareness and attitude of the company [67]. In the case of the SMEs in Balochistan Province, financial constraints were the main reason for their reluctance to invest in environmentally sustainable practices. While they could have sought financial help from personal sources, the complicated bank process and the prevailing business and security conditions made them hesitant to do so.  The positive mindset and attitude of the owners are crucial in driving environmental sustainability initiatives within the firm [38, 39]. SMEs, being owned and operated by their founders, can align their resources with their priorities, including prioritizing long-term sustainability over short-term profits [37]. If they see benefits in such environmental practices, they may allocate finances towards them, but if they view it as a long-term investment, they may be reluctant to adopt even if they have the means to do so [68]. The owners and managers may focus on certain sustainability aspects while disregarding others, depending on their mentality [69]. However, some owners may only consider sustainability issues when they face problems and otherwise prioritize profits over sustainability [66, 67].  The limited knowledge of SMEs owners and managers may lead them to believe that pro-environmental measures are costly without considering the long-term benefits, such as the reduction of toxic waste and energy consumption through eco-friendly machinery and practices like using energy-saving light fixtures [70]. Researchers contended that further incentives are needed to convince SMEs owners to take bolder pro-environmental measures, even if they have already taken initial steps [71]. Moreover, the study highlights that religion plays a role in shaping attitudes toward environmental sustainability. Nearly all religions teach similar moral values, but the intensity and nature of belief may vary [72]. Researchers showed a correlation between religion and environmental sustainability [73], where owners in Balochistan agreed that religion teaches ethical values, but finances, lack of education, and knowledge limit the practical application of these values.  These findings are in collaboration with previous studies that support a positive correlation between environmental awareness and practices among SME owners/managers [22, 52, 55]. This study suggests that SMEs may struggle to implement environmental management practices due to a lack of awareness of their environmental impact. Moreover, environmental awareness is influenced by legislation, leading to formal compliance systems and changes in conservation behavior within SMEs [53]. The majority of SMEs owners and managers lack the education and research environment to adopt pro-environmental practices, and their attitudes are shaped by media exposure. Some established SMEs practice corporate social responsibility, while others view it as separate from profit-making or as a burden on their income. |

|  | Reviewer 3 Comment | Author's Response |
| --- | --- | --- |
| 1 | The topic is important and timely. The methodology is unique as well but need further improvement in all sections to meet the standard of quality publication. | Thank you for your comments. We have improved the article by following your instructions. |
| 2 | Abstract is comprehensive. | Thank you for your comment. |
| 3 | “SMEs can play a significant role in promoting environmental sustainability,”..provide justification that why SMEs are deserving priority over large firms? | Thank you for your comments. We have improved the introduction by following your suggestions.  Page 1, Lines 30-36  SMEs have not received significant attention in the ongoing global discussion about sustainable development [4, 5]. It is evident that large organizations impact environment. It is also crucial to recognize the role of SMEs as well. SMEs impact on environment become clearer when we consider their collective impacts. Globally, over 95% of businesses are SMEs [6]. Although each SME may have little impact, the cumulative effect of SMEs can be substantial due to their large number [4]. It is often mentioned that SMEs are responsible for major pollution [7].  Page 1, Lines 40-45; Page 2, Lines 1-7  Researchers have been supporting the adaptation of sustainability practices [11, 3]. However, literature has not adequately addressed the sustainability issues faced by SMEs in developing countries as it has with developed ones [12]. This situation has been widely acknowledged in the literature [4, 5]. Wang et al. [10] shed light on the importance of sustainability initiatives in developing countries and the unique barriers and motivational factors. Similarly, Jabbour et al., [4] emphasized the significance of sustainability in developing countries and the need to study strategies for achieving sustainable practices. Additionally, Purwandani & Michaud [13] recognized the necessity to delve into sustainability matters within SMEs as it impacts business performance and presents new market opportunities.  Page 2, Lines 14-27  In this study we address the gap by exploring the internal and external factors that contribute to achieve the environmental sustainability in SMEs of Pakistan. The present study recognizes the existence of environmental alerts in Pakistan, particularly in Balochistan that comprise great environmental concern and weak governance [15, 16, 17]. Hence, this research significantly contributes to the discipline of sustainability. Further, the research framework of this study encompasses environmental orientation, responsible environmental management, and eco-friendly practices, which are grounded on strategic competencies such as pollution prevention, product stewardship, and sustainable development of natural resource-based view (NRBV) theory [18, 19]. Therefore, this study provides comprehensive understanding of internal and external dimensions of SMEs that need to be nurtured to improve environmental sustainability. While extending the previous research Baah et al. [18], this research provides new insight for top management, policymakers, governmental and non-governmental organizations (NGOs) for achieving environmental performance in SMEs of developing countries |
| 4 | In the literature review researcher highlighted the internal and external factors nicely however I believe there are more external factors can be incorporated apart from stakeholder’s aspects such as community/societal influence, buyers pressure, market dynamics and so on. Internal barriers part also can be enhanced. I highly encourage author (s) to read this paper to improve these lacking.  -Hossain, M. I., Ong, T. S., Tabash, M. I., Siow, M. L., & Said, R. M. (2022). Systematic literature review and future research directions: drivers of environmental sustainability practices in small and medium-sized enterprises. International Journal of Sustainable Economy, 14(3), 269-293. | Thank you for your comment. We have improved the literature review and incorporated your instructions on page 2, lines 44-47; page 3, lines 1-50; page 4, lines 1-21; page 5, lines 1-45; page 6, lines 1-27. Page 2, Lines 44-47; Page 3, Lines 1-50; Page 4, Lines 1-21; Page 5, Lines 1-45; Page 6, Lines 1-27 SMEs prioritize survival and cost reduction that may not align with environmental sustainability [25]. The relationship between environmental sustainability and economic outcomes is complex and influenced by multiple factors. However, engaging in environmentally efficient practices can lead to long-term benefits and increased revenue through recycling, reuse and reduce [26]. Hence, it raised question whether eco-efficiency could increase profits for SMEs [27]. Researchers suggest that adaptation of sustainable practices and ecological partnerships can lead to financial success for SMEs [28]. Moreover, Hossain et al. [29] conducted a systematic literature review of articles published during 2009–2020 and identified 87 drivers of environmental sustainability and categorized them under internal and external dimensions. Researchers suggested that SMEs should focus on internal and external factors for implementation of sustainable practices. Hence, researchers identified internal and external barriers suitable to the context of this study. Table 1 summarizes the barriers and opportunities for SMEs. In reality, adopting sustainability requires resources, however, SMEs already facing several challenges [30]. On the other side, SMEs strive to gain flexibility and adaptability [31], unfortunately SMEs often lack the resources to implement sustainable practices [10]. Researchers argue that factors such as time constraints, financial limitations, inadequate training, information access and a lack of environmental management are hindering SMEs in practicing environmental sustainability [13, 32]. A limited number of staff and routine work leave no time for learning and implementing sustainability measures [32]. Hiring professionals to train existing employees can help, however, financial constraints restrict SMEs to invest in knowledge and ability enhancement. Another aspect is employee or manager reluctance towards green innovation due to tight schedule, lack of experience or latest knowledge [33]. Environmental practices require pro-environmental behavior of employees [34]. To address this problem, companies should establish a network and enhance their relationship with external partners [35], such as universities, to provide cost-effective training for their staff. Establishing short-term objectives may facilitate the achievement of long-term goals, allowing SMEs to foster internal sustainability. The limited financial capital of SMEs creates a significant constraint in SMEs ability to invest in environmental sustainability practices. The impact of these investments on profits remains a lingering question. Despite the potential benefits, SMEs may struggle to sustain the financial burden of implementing environmental sustainability measures [36]. Consequently, SMEs can apply for grants and loans from government agencies and financial institutions to alleviate financial constraints, which, in return, help them to bring sustainability in process and increase revenue in long term. However, lack of financial support from government and financial institutions and formal lengthy procedures creates hindrances in adopting sustainable practices [13, 37]. Despite the awareness and willingness of owners and managers to embrace environmental sustainability, financial constraints prevent them from environmental initiatives or involving third parties [38]. Moreover, the decision to adopt environmental sustainability practices in SMEs lies with the owners and managers, who act as the decision-makers. Their perception of the benefits associated with pro-environmental behavior is a key factor in determining whether they choose to invest in such practices [34]. A positive attitude towards environmental sustainability is essential for owners and managers to initiate such practices in their firms [38, 39]. Manager’s age determines the adaptation of sustainable practices and concerns for environmental performance [40]. Education and green entrepreneurial orientation also play a significant role in adopting sustainable practices in SMEs [35]. SMEs are mostly governed by their owners and family members hence they align their resources with their priority, and survival is the topmost priority. Thus, sustainability may be considered on priority in SMEs when owners, family member or managers have environmental instincts and can foresee that bringing sustainability is new way of survival. On the other hand, the interests of stakeholders play a significant role in firm's approach towards environmental sustainability at different levels within the business [41]. Stakeholders often prioritize financial sustainability over social and environmental sustainability, as long as they receive some sort of return [42]. Large organizations can establish long-term partnerships with stakeholders and the resources to influence through marketing strategies. Albeit SMEs find it difficult to maintain the relationships in long-term and often have shorter-term relationships with stakeholders, thus making it difficult for SMEs to change their perceptions [25]. SMEs are supposed to have a responsibility to the community, customers, and employees they serve [37, 38, 43]. Jamali et al. [44] argue that SMEs operate on a personal level and are closer to their stakeholders due to work-family issues, trust, and employee retention, among other factors. Moreover, when owners see market forces and customers direction towards eco-friendly processes and products, they may shift their focus on sustainability. Researchers claim that owners may not consider sustainability issues until they face a problem and are not motivated to do so as long as they are reaping profits [45]. **Table 1. Barriers and opportunities for SMEs.** **Barriers For SMEs** \| **Opportunities for SMEs**; - Insufficient knowledge among owners/managers or employees. - Limited workforce size. - Constrained financial resources. - Inadequate organizational capacity. - Restricted and short-term business operations integrated into daily activities. - Pessimistic belief that engaging in environmental sustainability practices might have a prolonged payback period. - Non-compliance or lack of adherence to government legislation. - Concerns about potential temporary cost increases for products, with owners fearing customer resistance to higher prices. - Different levels of awareness among owners/managers. - Diverse attitudes among owners/managers. - Reluctance of owners/managers to alter their perspectives on environmental sustainability practices. - Lack of awareness among stakeholders regarding the advantages of environmental sustainability practices compared to potential price increases. - Limited knowledge about technological advancements and their potential benefits for overall SME performance. \| - Implementation of laws and regulations mandating SMEs to adopt environmental sustainability practices. - Flexibility in altering the existing setup. - Proximity and strong ties with suppliers. - Commitment of owner management towards incorporating environmental sustainability practices. - Introduction of green innovative products. - Utilization of part-time employees to align with new practices. - Enhanced awareness about the benefits of environmental sustainability practices. - Influence from community expectations regarding environmental sustainability. - Increasing interest from investors in supporting and promoting environmental sustainability practices. **Environmental sustainability practices and firm performance** The incorporation of sustainable practices in SMEs is influenced by various factors [10, 13]. These factors include the motivation of entrepreneurs to adopt certain practices based on their perception of potential positive financial outcomes or their concerns about the consequences of not embracing eco-friendly approaches [46]. The actions taken towards sustainability can either positively or negatively impact the financial performance of SMEs. Some researchers suggested that implementing sustainability practices is considered an additional burden by SMEs [47], while others support that there is positive relationship between firm performance and environmental protection efforts [8, 36]. Environmental Management System (EMS) has proven to be highly beneficial for the sustainable development of SMEs. ISO 14001 offers certified guidelines to assist SMEs in reducing their environmental impact and achieving long term sustainability [48]. By implementing EMS, SMEs can lower energy consumption costs while also creating positive image in the eyes of customers, regulators, and the public. Furthermore, EMS enables SMEs to utilize latest green technology, which not only reduces costs and improves productivity but also minimizes their negative impact on the environment. This innovative approach to resource utilization, cost reduction and environmental preservation ultimately ensures the sustainability of SMEs [24, 49]. Additionally, when SMEs collaborate with businesses within their network and receive support from organizations, they become more environmentally innovative and effectively reduce costs [22]. This ability to leverage network advantages is crucial for SMEs. Subsequently, adoption of environmental practices allows SMEs to position themselves as excellent performers [50]. Researchers suggested that financial limitations were inversely related to the adoption of practices. However, it also revealed a correlation between access to capital and technological expertise with the implementation of sustainability measures regardless of company size [50]. Various approaches can be utilized to assess the impact of environmental sustainability practices on firm performance. These include enhancing stability, reducing emissions , improving eco-efficiency systems, conserving materials, energy and implementing ISO 14001 certification [9, 8, 24, 48]. Performance measures may involve creating environmentally friendly production processes and adopting innovative techniques. By adopting environmental sustainability practices firms can enhance their systems to minimize water usage, air pollution and toxic waste emissions [51]. Therefore, managers of SMEs who implement sustainability measures often believe in adopting innovative technologies [52]. The positive impact of effectively implemented EMS, on performance has been evident [48, 53]. Therefore, implementation of EMS leads to improved environmental performance, including reductions in water consumption and waste emissions. Previous studies found that the adoption of EMS practices provides benefits to SMEs [53]. Improvements in environmental performance and cost reduction go hand in hand. Eco-design plays a role in creating products with better recycling potential and improved performance. Furthermore, these eco-friendly designs are not only less harmful but also cost-effective to manufacture, leading to reduced expenses and improved firm performance [54]. When organizations strive to reduce pollution, it ultimately results in resource utilization and increased productivity. Sustainable practices like recycling and collaborating with suppliers contribute to cost reduction and enhanced efficiency. By utilizing metal scraps, waste materials, and recycled oil within SMEs operations along, with water recycling and purification measures, all contribute to cost reduction while simultaneously boosting profits. Furthermore, implementing environmental protection initiatives can influence firm image [54]. Firms’ performance heavily relies on their knowledge-based resources and the ability to effectively utilize these resources to gain advantages [52]. However, researchers argue that being an eco-friendly organization does not necessarily guarantee strong competitiveness in the market [50]. **Conceptual framework** This study proposes a research model to investigate sustainability in SMEs. Previous studies have examined how internal and external factors affect knowledge and attitudes toward environmentally conscious management practices [55]. This study included external and internal factors as independent variables, which encompasses suppliers, customers, and laws. These characteristics motivate and challenge management to adopt environmental sustainability initiatives. Stakeholder impact on SMEs is further influenced by environmental awareness and attitudes, which address top management understanding of the cost and benefits of being environmentally sustainable. These variables influence environmental sustainability practices, serving as dependent variable. Hence, external and internal factors influence the organization's attitude and understanding of environmental sustainability, which defines its policies, strategies, and systems for sustainable development. This process is influenced by owner-manager qualities, time, financial resources, and cost-benefit information about environmental sustainability. Without these criteria, SMEs may struggle to implement environmental sustainability initiatives. The effects of these factors need to be examined in the context of developing countries. Fig 1 depicts the research framework. ` **External** **Factors** **Environmental Awareness and Attitudes** **Environmental Sustainability Practices** **Internal** **Factors** **Fig 1. Conceptual framework.** |
| 5 | Why the scope chosen only Balochistan? Justify. And importance of the research on Baluchistan needs to incorporate in introduction section, why it’s so crucial and urgent. | Thank you for your comment. We have incorporated your suggestion in the introduction section on page 2, lines 14-18.  In this study we address the gap by exploring the internal and external factors that contribute to achieve the environmental sustainability in SMEs of Pakistan. The present study recognizes the existence of environmental alerts in Pakistan, particularly in Balochistan that comprise great environmental concern and weak governance [15, 16, 17]. Hence, this research significantly contributes to the discipline of sustainability. |
| 6 | “Due to the sensitive nature of the topic and the underdeveloped environment in Balochistan, the sample size was limited to 30 SMEs.”…these are not well convincing reason …how about the saturation point, did that met and how you ensure that no need further data collection? | Thank you for your comment. We have provided references for the appropriate sample size for qualitative study on page 8, lines 9-12.  Page 8 Lines 9-12  Prior studies recommended a sample size of 12–20 for qualitative research [59, 60]. Hence, the sample size of this study is deemed acceptable [61]. |
| 7 | Provide definition of SMEs in your context in methodology section. | Thank you for your comment. We have provided the definition of SMEs on page 6 lines 29-30.  Page 6 Lines 29-30  The target population for this study was SMEs, defined as firms with fewer than 250 employees [56], located in Balochistan, Pakistan |
| 8 | 4-star hotel is this a SME? Confirm. That’s why you need to mention the criteria of choosing SMEs. | Thank you for your comment. We have corrected the typographical error. |
| 9 | Mention the question asked in the interview.  The quotation from informants needs to add (not all quotation and no need full only key statements) where suitable otherwise it shows only researcher's opinion. | Thank you for your comments. We have included the interview questions in Table 3. Furthermore, we have incorporated the respondents' answers into the results. |
| 10 | Better to have a graphical overview/model of nodes/codes and emerged themes. | Thank you for your comments. We have included word clouds and a table of emerged themes, as given below;  **Table 4. Emerged themes.** **Themes** \| **Sub-themes**; General Overview of SMEs' Environmental Awareness \| Level of Education; \| Sound knowledge; \| Lack of education; \| International Business Impact; Perception of Environmentally Friendly Practices \| Long-Term Investments; \| Conservation; External Factors Impacting Sustainability Practices \| Influence of international customers on environmental policies; \| Challenges with environmentally friendly practices; \| Government involvement in promoting sustainability; \| Adoption of sustainable practices; Internal Factors Impacting Sustainability Practices \| Scale of Business; \| Varied business performance across sectors; \| Challenges in accessing credit and loans; \| Training and development; \| Lack of financial resources for environmentally safe machinery; Impact of Awareness and Attitudes on Sustainability Practices \| Influence of education on understanding sustainability; \| Different views on the relationship between corporate social responsibility and profit; \| Willingness to adopt environmentally friendly practices for long-term sustainability; \| Religious and ethical perspectives on environmental responsibility; Environmental Practices (Policies, Strategies, Systems) \| Presence and nature of environmental policies; \| Resource conservation and waste material reuse efforts; \| Inclusion of environmental perspective in employee training; \| Participation in voluntary environmental programs; \| Challenges faced in implementing sustainability practices |
| 11 | “Awareness and attitude of the company owners and managers play a critical role in determining the level of engagement in environmentally sustainable practices.” Suggest how to enhance the level of engagement.  Managerial implication need to be aligned with the findings of the study rather than just mention generic statements. | Thank you for your comments. We have enhanced the managerial implications by incorporating your feedback on page 19, lines 19-41.  Page 19 Lines 19-41.  The study provides significant implications for SMEs owners, managers, government, and environmental agencies. First, it is utmost important for both owners and workers to have access to education regarding environmental initiatives and their advantages. Closing the knowledge gap through workshops and training programs is essential, not only for aware SMEs but also to make them stick to eco-friendly protocols. Additionally, government agencies and environmental NGOs should advocate for increased government support and incentives as financial limitations pose a barrier. Second, SMEs should view ISO certification not as a requirement but also as a strategic tool that provides an advantage in accessing international markets. SMEs should engage with customers to raise awareness about the benefits of eco-friendly practices, which can help address customers’ concerns about increased prices. Third, SMEs should position themselves as environmentally friendly organizations that open opportunities for financial support mechanisms such as environmental initiative grants and loans. Fourth, managers should develop CSR strategies that align with SMEs priorities, which create shared value and enhance reputation. Fifth, managers should collaborate with other environmental agencies and establish networks with NGOs and governmental agencies to amplify the impact of sustainability initiatives. Managers should also explore the opportunities for adaptation of climate friendly technologies and processes. Sixth, government agencies should take initiatives to foster a mindset among SMEs owners by emphasizing how sustainability aligns with long term profitability. Finally, government agencies should develop policies to monitor and evaluate environmental initiatives and performance of SMEs, which ensures improvement and adaptation to environmental initiatives. These research implications contribute to long term success and sustainable development of SMEs. |
| 12 | Conclusion section needs to be more streamed and concise. | Thank you for your comment. We have improved the conclusion section as per your instructions on page 19, lines 42-46 and page 20, lines 1-13.  This study examines the impact of internal and external factors on the environmental sustainability of SMEs in Balochistan, Pakistan. The qualitative research method was used to gain a deeper understanding of the motivators and barriers towards environmental sustainability. This study indicated that adoption of environmental sustainability practices by SMEs is influenced by a combination of internal and external factors. Further, awareness and attitude of the company owners and managers play a critical role in implementation of environmentally sustainable practices. Internal factors, such as financial constraints and lack of awareness, were identified as the primary reasons for the limited adoption of environmentally sustainable practices among SMEs in Balochistan province. However, the study also found that applying for grants and loans from government agencies or non-government organizations can help SMEs overcome financial constraints and pursue environmental sustainability. Further, external factors, such as customers and government policies, play a crucial role in promoting environmental sustainability among SMEs. In the similar vein, these internal and external factors significantly influence eco-friendly attitude and awareness of SMEs owners or managers, which leads to overcome barriers such as lack of knowledge, polices, training, and foster positive attitude for adoption of environmental sustainability practices within SMEs. |
| 13 | Pls consult with professional proof-reader to improve the language quality. | Thank you for your comment. We have proofread the manuscript. |

We hope you will appreciate our efforts. In case you feel that we have missed something out or failed to execute your suggestions, we are prepared to take further action.

Yours sincerely,

Corresponding author on behalf of all co-authors
